# Supplementary material for: The interactome of intact mitochondria by cross-linking mass spectrometry provides evidence for coexisting respiratory supercomplexes
Source: Mol Cell Proteomics. 2017 Dec 8;17(2):216–32. doi: 10.1074/mcp.RA117.000470 (PMC5795388; doi:10.1074/mcp.RA117.000470)
Supplement: Supplemental Data [file supp_RA117.000470_133922_0_supp_23978_fzffwf.zip › spectra_annotation/mito_DR_spectra_annotation/259-1-1-1-4-1.pdf]

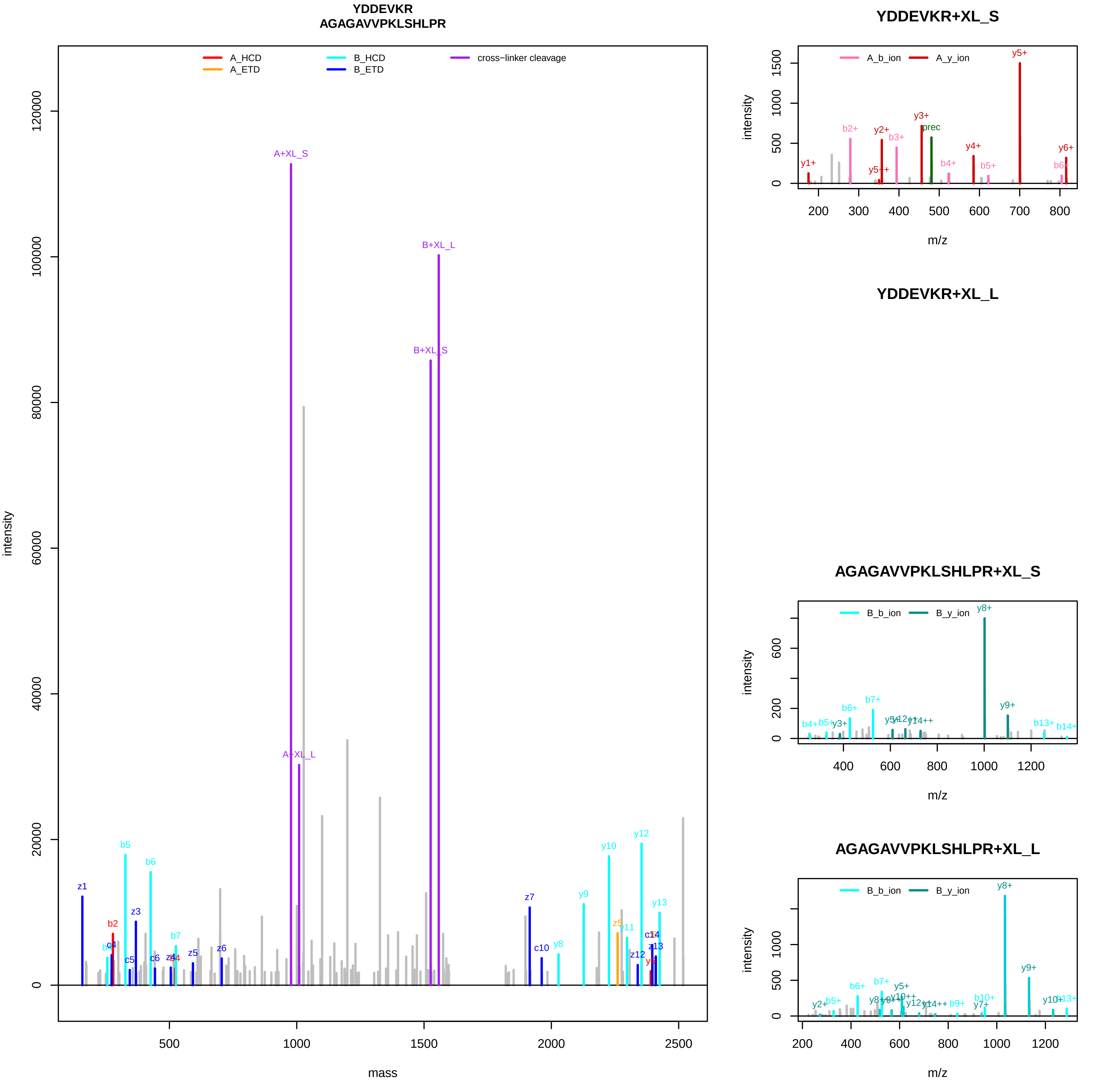
YDDEVKR
AGAGAVVPKLSHLPR
A_HCD
A_ETD
B_HCD
B_ETD
cross−linker cleavage
A+XL_S
B+XL_L
B+XL_S
A+XL_L
z1
b2
b5
b6
z3
c4
c5
c6
b7
z5
z6
z7
c10
y8
y9
y10
y11
y12
y13
z5
z12
c14
z13
mass
intensity
0
20000
40000
60000
80000
100000
120000
500
1000
1500
2000
2500
YDDEVKR+XL_S
A_b_ion
A_y_ion
y1+
b2+
y2+
b3+
y3+
prec
y4+
y5+
y6+
b4+
b5+
b6+
y5++
m/z
intensity
YDDEVKR+XL_L
AGAGAVVPKLSHLPR+XL_S
B_b_ion
B_y_ion
b4+
b5+
y3+
b6+
b7+
y8+
y9+
b13+
b14+
m/z
intensity
AGAGAVVPKLSHLPR+XL_L
B_b_ion
B_y_ion
y2+
b5+
b6+
b7+
y5+
y8+
y9+
b10+
y10+
b13+
m/z
intensity
